# Secondary Metabolites Profiling, Antimicrobial and Cytotoxic Properties of *Commiphora gileadensis* L. Leaves, Seeds, Callus, and Cell Suspension Extracts

**DOI:** 10.3390/metabo13040537

**Published:** 2023-04-10

**Authors:** Ayed M. Al-Abdallat, Batool K. Adayileh, Jamal S. Sawwan, Rida Shibli, Tamara S. Al-Qudah, Bashaer Abu-Irmaileh, Randa N. Albdaiwi, Jehad Almaliti, Yasser Bustanji

**Affiliations:** 1 Department of Horticulture and Crop Science, School of Agriculture, The University of Jordan, Amman 11942, Jordan; b.adaileh@ju.edu.jo (B.K.A.); jsawwan@ju.edu.jo (J.S.S.); 2 Department of Agricultural Biotechnology and Genetic Engineering, Faculty of Agriculture Technology, Al-Ahliyya Amman University, Amman 19328, Jordan; r.shibli@ammanu.edu.jo; 3 Hamdi Mango Center for Scientific Research, The University of Jordan, Amman 11942, Jordan; t.alqudah@ju.edu.jo (T.S.A.-Q.); b.aburmela@ju.edu.jo (B.A.-I.); 4 Faculty of Science, Zarqa University, Zarqa 13110, Jordan or randa.rahahla@ju.edu.jo; 5 Department of Land, Water and Environment, School of Agriculture, The University of Jordan, Amman 11942, Jordan; 6 Department of Pharmaceutical Sciences, School of Pharmacy, The University of Jordan, Amman 11942, Jordan; j.almaliti@ju.edu.jo; 7 Scripps Institution of Oceanography, University of California, San Diego, CA 92093, USA; 8 Department of Basic Medical Sciences, College of Medicine, University of Sharjah, Sharjah P.O. Box 27272, United Arab Emirates; 9 Department of Biopharmaceutics and Clinical Pharmacy, School of Pharmacy, The University of Jordan, Amman 11942, Jordan

**Keywords:** antimicrobial, Burseraceae, callus, cell suspension, *Commiphora gileadensis*, cytotoxicity, LC-MS

## Abstract

*Commiphora gileadensis* L. is an important endangered medicinal plant that belongs to the family Burseraceae. In this study, *C. gileadensis* callus culture was established successfully using mature leaves as explants cultured on Murashige and Skoog (MS) media supplemented with 24.50 μM of indole butyric acid (IBA) and 2.22 μM 6-Benzylaminopurine (BAP) (callus induction media). The obtained callus was maintained on MS medium supplemented with 16.11 μM naphthalene acetic acid (NAA) in combination with 6.66 μM BAP, which resulted in a substantial increase in callus fresh and dry weights. The cell suspension culture was established successfully using liquid callus induction media supplemented with 3.0 mg·L^−1^ proline. Thereafter, the chemical constituents of different *C. gileadensis* methanolic extracts (callus, cell suspension, leaves, and seeds) were profiled, and their cytotoxic and antimicrobial properties were investigated. The LC-MS GNPS analyses were applied for chemical profiling of the methanolic plant extracts, and several natural products were identified, including flavonols, flavanones, and flavonoids glycosides, with two unusual families that included puromycin, 10-hydroxycamptothecin, and justicidin B. The methanolic extracts have shown selective antimicrobial and cytotoxic properties against different microbes and cancer cell lines. For instance, leaf extract showed the highest zone of inhibition for *Staphylococcus aureus*, while cell suspension culture was effective against *Staphylococcus epidermidis* and *Staphylococcus aureus.* All extracts showed selective activity against A549 cell lines for the cytotoxicity assay, while the leaf extract had a broad cytotoxic effect against all tested cell lines. This study revealed that *C. gileadensis* callus and cell suspension cultures can be employed to increase the in vitro formation of biologically active compounds that may have cytotoxicity and antibacterial action against different cancer cell lines and bacterial species. Further studies are required to isolate and identify such constituents that corroborate the observed activities.

## 1. Introduction

*Commiphora gileadensis* L. (common name: Arabian balsam, Mecca myrrh, opobalsam, or biblical balm of Gilead) is a wild medicinal plant that belongs to the family Burseraceae [1]. The plant can be described as a small dioecious shrub, almost two to four meters in height, with a dark green stout trunk, papery thin peeling bark with an alternative fascicle, and compound leaves carried on small lateral shoots [2]. It is naturally distributed in eastern Ethiopia, eastern Sudan, Somalia, Oman, Yemen, and Saudi Arabia. The plant was cultivated in ancient times in the Jordan Valley, close to the Dead Sea region, before its extension 1000 years ago, as documented in the Madaba mosaic map [3]. The plant is currently disappearing from its natural habitat due to overgrazing, overuse by indigenous populations, and climate change-associated conditions [4].

*C. Gileadensis* is considered an important plant because of its economic value [1]. It produces many secondary metabolites and volatile oils that can be used in the medicine and perfume industry [5]. It was used widely in ancient times in religious virtues, the treatment of many diseases, and it is considered a pain killer and an excellent wound healer due to its beneficial properties on human skin [6]. *C. gileadensis* has been used in Ayurvedic medicine due to its therapeutic effects against inflammatory diseases, coronary artery diseases, gynecological diseases, and obesity [7]. The extracts of *C. gileadensis* have shown antioxidant, antimicrobial, antiviral, antidiabetic, poison antidotes, protective effects against confluent necrosis in the liver, and cytotoxicity activities. Moreover, it reduces the in vitro growth of prostate, liver, and other types of cancer cell lines [8,9,10].

The medicinal properties of *C. gileadensis* are mainly attributed to the presence of several active components extracted from its stem, bark tissues, flowers, and leaves [7,11]. These bioactive compounds include β-pinene, β-caryophyllene, and eugenol [12]. The oil extracts of *C. gileadensis* contain several terpenoids, such as terpinene-4-ol, δ-cadinene, commigileadin, canophyllal, and flavonols such as quercetin and mearnsetin [13]. Recently, high-performance liquid chromatography-tandem mass spectrometry (HPLC/MS^n^) was used to identify several active compounds from butanol-extract of *C. gileadensis* stem bark that included phenolic compounds, flavonoids, prenylated flavonoids, coumarin, an alkaloid, and chalcone [14].

In the *Commiphora* genus, which includes approximately 190 species, 21 of these species are currently listed in the International Union for Conservation of Nature’s (IUCN) Red List of Threatened Species. Several methods were applied to conserve these plants; unfortunately, most of these methods didn’t succeed in conserving these plants in their natural habitats [15]. Therefore, there is an urgent need to develop new methods to conserve and utilize *Commiphora’s* genetic resources without negatively affecting their number in natural habitats. Furthermore, such methods can be used to exploit and boost secondary metabolite production using unconventional approaches [16]. In this perspective, callus and cell suspension cultures are considered promising in vitro systems for secondary metabolite production from endangered plants [17]. For example, many secondary metabolites were extracted through in vitro culture techniques, such as the biosynthesis of guggulsterone (a hypolipidemic natural agent produced in resin canals) from cell suspension cultures of *C. wightii* [18]. Therefore, the establishment of callus and cell suspension culture systems of *C. gileadensis* will be of great importance for germplasm conservation, micro-propagation, and secondary metabolite production.

In this study, we described the establishment of *C. gileadensis* callus and cell suspension cultures for the massive production of secondary metabolites in vitro. Methanolic extracts from callus and cell suspension cultures, as well as naturally grown leaves and seeds, were prepared, and their chemical compositions were then investigated using liquid chromatography-tandem mass spectrometry (LC-MS/MS) coupled with mass spectral molecular networking analysis using the Global Natural Products Social Molecular Networking (GNPS) platform. Finally, the cytotoxic and antimicrobial activities of *C. gileadensis* callus and cell culture methanolic extracts were evaluated and compared to extracts from different plant parts.

## 2. Materials and Methods

### 2.1. Plant Material

A *C. gileadensis* plant was obtained as a kind gift from the Oman Botanic Garden/Oman Authority of Environment/The Sultanate of Oman (voucher number: JU-OBG-CG#2018.1). For the establishment of the callus culture, mature leaves were collected and used as explants. In addition, different plant parts (leaves and seeds) were used for the cytotoxicity and antimicrobial assays.

### 2.2. Callus Culture Establishment and Maintenance

To establish *C. gileadensis* callus culture, fully mature leaves were excised from the plants and washed using a mild detergent with running tap water for 15 min. The explants were then surface sterilized by soaking in a 0.1% (*w*/*v*) mercuric chloride (HgCl_2_) solution for 2 min and rinsing three times in sterile distilled water before use.

For callus induction, a single sterilized leaf with an attached petiole was used as an explant. Murashige and Skoog (MS) media [19] supplemented with 0.1 M sucrose, 100 mg·L^−1^ myo-inositol, and 16 different combinations of Indole Butyric Acid (IBA) (2.45, 6.13, 12.25, 24.50 μM) and 6-Benzylaminopurine (BAP) (2.22, 5.55, 11.10, 22.20 μM) were tested for callus induction. The pH of the media was adjusted to 5.8 before adding 6 g·L^−1^ agar (Bacto-agar, Difco, India), then the media was autoclaved and dispensed as 25 mL aliquots into Petri dishes, and thereafter the sterilized explants were placed directly onto assigned callus induction media. A Complete Randomized Design (CRD) was applied, and each treatment was replicated three times where each replicate consisted of three explants in a single Petri dish. The cultures were then incubated at 24 °C under complete darkness and monitored for callus induction for eight weeks. After callus induction, ~0.5 g of callus was transferred into 250 mL flasks containing 50 mL of MS medium supplemented with the best IBA and BAP combination for callus induction (24.50 μM IBA and 2.22 μM BAP). The cultured callus was maintained under complete darkness at 24 °C. Induced calli were then subcultured on the same medium for up to six intervals, and after having enough callus materials as mother stock, further experiments were conducted.

To test the effect of different combinations of Naphthalene Acetic Acid (NAA), as an auxin source, and BAP, as a cytokinin source, on callus growth and development, 0.5 g of callus was subcultured on different MS solid media supplemented with 0.1 M sucrose, 100 mg·L^−1^ myo-inositol, and different concentrations of NAA (0, 8.06, and 16.11 μM) in combinations with different concentrations of BAP (0, 6.66, and 13.32 μM) and the best callus induction media (24.50 μM IBA and 2.22 μM BA) was used as a control. Treatments were incubated for eight weeks under complete darkness at 24 °C, and at the end of the experiment, callus freshness and dry weight (oven-dried for 24 h at 50 °C) were measured. A CRD was used, and each treatment was replicated three times, and each replicate consisted of three explants in a single Petri dish. The medium with the best combination of NAA and BAP that resulted in the best callus growth was renamed “callus maintenance medium” and used to maintain callus culture by subculturing ~0.5 gm of callus on it every eight weeks. 

For callus induction and callus production experiments, the obtained data were analyzed using SAS software (version 9, SAS Inc., Cary, NC, USA), the analysis of variance (ANOVA) was obtained, and mean separation was performed using Tukey’s HSD test at a probability level of 0.05.

### 2.3. Cell Suspension Establishment and Maintenance

To establish *C. gileadensis* cell suspension culture, 50 mL of MS liquid media supplemented with 0.1 M sucrose and 100 mg·L^−1^ myo-inositol with either 24.50 μM IBA and 2.22 μM (best callus induction medium) or 16.11 μM NAA and 6.66 μM BAP (the best callus maintenance medium) were dispensed into 250 mL capped flasks. In addition, both media types were supplemented with either 0.0 or 3.0 mg·L^−1^ of proline, and a liquid MS medium free of plant growth regulators was used as a control. Thereafter, 1 g of friable *C. gileadensis* callus (collected from the callus maintenance medium) was cut into very small pieces and suspended in the liquid MS media. Capped flasks were then placed on an orbital shaker at 50 rpm under the same conditions described for callus induction experiments. The best cell suspension growth medium (24.50 μM IBA + 2.22 μM BAP) supplemented with 3.0 mg·L^−1^ proline was used to establish the mother stock of the cell suspension. Cultures were subcultured many times until enough cell suspension culture was obtained to use in further experiments.

Cell growth was monitored in liquid MS medium by recording the fresh weight of harvested cells every three days up to three weeks in culture. A CRD was used, and each treatment was replicated five times, with each replicate consisting of one flask for each time point. Data were analyzed using SAS software (version 9, SAS Inc., Cary, NC, USA). and the analysis of variance (ANOVA) was obtained. The mean separation was calculated using Tukey’s HSD test at a probability level of 0.05. 

### 2.4. Preparation of Methanolic Extracts

Leaf and seed samples that were obtained from the wild plant and callus and cell suspension culture samples were used for methanolic extract preparation. The samples were air-dried at room temperature in the shade until they reached a constant weight, at which point they were grounded and weighed. The extraction was carried out using methanol (HPLC grade methanol (99.9%); Sigma-Aldrich, St. Louis, MO, USA) based on dry weight with a ratio of 1:10 (1.0 g of sample in 10 mL solution), and the extract solutions were left for three days with continuous stirring. A rotary evaporator (RE300 rotary evaporator; Stuart Vacuum Pump, Staffordshire, UK) was then used to evaporate the extracts until they were completely dry. The dried extracts were then dissolved in Dimethyl sulfoxide (DMSO: Fisher Scientific Company, UK), whose volume was determined by the following equation:

DMSO (mL) = weight of evaporated extract × 10); to achieve a concentration of 100 mg·L^−1^

### 2.5. Chemical Constituents and Molecular Networking Analysis 

The chemical constituents of *C. gileadensis* callus and cell suspension and leaf methanolic extracts were analyzed using an untargeted approach through liquid chromatography-tandem mass spectrometry (LC-MS/MS). The LC-MS/MS was coupled with emerging MS-based computational analysis approaches to identify new families of natural products within *C. gileadensis* methanolic extracts. In brief, LC-MS/MS data of *C. gileadensis* samples were subjected to mass spectral molecular networking analysis using the Global Natural Products Social Molecular Networking “GNPS platform” as described previously [20]. In molecular networking, all identical MS/MS spectra are grouped to give a list of unique MS/MS spectra. These were then subjected to spectral alignment, enabling spectral matching with offsets based on the precursor mass differences. Molecules generating similar MS/MS spectra were bundled due to similarities in their fragmentation patterns and were then referred to as molecular families. A molecular family was defined as a set of MS/MS spectra that are structurally related and have some similarities. In addition, the MS/MS spectra were putatively annotated against reference spectra within the Global Natural Products Social Molecular Networking (GNPS) platform [20]. The reference libraries that can be searched because their spectra are publicly available include: NIST17, Massbank Europe and North America, ReSpect, Critical Assessment of Small Molecule Identification (CASMI), the European Molecular Biology Library (EMBL) metabolomics library, the Human Metabolome Database (HMDB), and GNPS contributed MS/MS spectra. The resulting molecular networks visualized the chemical relationships of compounds and provided a unique and important tool for detailed interpretation of chemical revolutions.

The following parameters were applied for the network analysis using GNPS: A molecular network was created using the online workflow at GNPS. The data were filtered by removing all MS/MS peaks within ±17 Da of the precursor m/z. MS/MS spectra were window filtered by choosing only the top six peaks in the ± 50 Da window throughout the spectrum. The data were then clustered with MS-Cluster with a parent mass tolerance of 2.0 Da and an MS/MS fragment ion tolerance of 0.5 Da to create consensus spectra. Furthermore, consensus spectra that contained less than two spectra were discarded. A network was then created where the edges were filtered to have a cosine score above 0.7 and more than three matched peaks. Further edges between two nodes were kept in the network if and only if each of the nodes appeared in the other’s respective top 10 most similar nodes. The spectra in the network were then searched against GNPS’ spectral libraries. The library spectra were filtered in the same manner as the input data. All matches kept between network spectra and library spectra were required to have a score above 0.7 and at least 3 matched peaks [21].

### 2.6. Antimicrobial Assays

The antimicrobial properties of *C. gileadensis* methanolic extracts were assayed on seven bacteria species: *Staphylococcus aureus* ATCC-25923, *Bacillus subtilis* ATCC-6633, *Staphylococcus epidermidis* ATCC-12228, *Salmonella* spp., *Escherichia coli*, *Erwinia carotovora,* and *Candida albicans* ATCC-10231, a pathogenic yeast species. Two different methods were used: the disc diffusion assay [22] and the minimum inhibitory concentration (MIC) assay (NCCLS, 2000), and for each assay, five replicates were used for each microbe type. For the disc diffusion assay, about 6 mm discs were made on the surface of Muller Hinton agar for the disc diffusion assay (Karlsmose, 2010); [22]. Then, about 100 μL of the bacterial suspension and 10 mg of the extract were loaded onto discs [22]. Thereafter, data were recorded according to the width of the inhibition zone at 37 °C in the incubator conditions, while for the MIC assay, data were recorded after 24 h for the lowest plant extract concentrations used to inhibit the microbe growth. The antibiotic tetracycline (10 mg·mL^−1^ for the diffusion assay) was used as a positive control, while the solvent dimethylesulfoxide DMSO was used as a negative control.

### 2.7. Cytotoxicity Assays 

The cytotoxic properties of *C. gileadensis* extracts were assayed by using several cell lines that included human breast adenocarcinoma (MCF-7), human epithelial pancreas carcinoma (PANC-1), human prostate adenocarcinoma, grade IV (PC-3), human epithelial lung carcinoma (A549), fibroblasts, and normal skin cells (CCD-1064SK). All these cell lines were obtained from ATCC. The A549 and PANC-1 cell lines were cultured in DMEM high glucose medium (Euro Clone), while MCF-7 and PC-3 were cultured in RPMI-1640 (DMEM, EuroClone) medium as recommended by ATCC. Both media were supplemented with 10% heated fetal bovine serum, 1% of 2 mM L-glutamine, penicillin (50 IU/mL) and streptomycin (50 µg·L^−1^). Fibroblast cells were cultured in Iscove’s medium as recommended by ATCC. The media were supplemented with 10% heated fetal bovine serum, 1% of 2 Mm L-glutamine, and (100 IU/mL), penicillin/(100 ug/mL) streptomycin. According to the cell’s growth profile, cells were seeded at a density of 7000 cells per well, while fibroblast seeding density was 1 × 10^5^ cells per well. Cell viability was determined by trypan blue exclusion using a hemocytometer as described previously [23].

For the cytotoxicity assay, cells were washed with phosphate buffered saline (PBS). The PBS was then decanted, and the cells were detached using 0.025% trypsin-EDTA (EuroClone). Thereafter, the proper media type for each cell line was added to a final volume of 10 mL. The cell suspension was then centrifuged at 1000 rpm for 10 min and the pellets were resuspended in a 10 mL medium to make a single-cell suspension. The viability of the cells was determined by trypan blue exclusion, and it exceeded 90% as counted in a hemocytometer. The cell suspension was then diluted afterward to give the optimal seeding density and 100 μL of the cell suspension was plated in a 96-well plate. Cells were cultured at 37 °C in a humidified atmosphere of 5% CO_2_ and after 24 h, the cells were treated with different concentrations of the *C. gileadensis* methanolic extracts. For this purpose, each extract was diluted with the medium to give different concentrations (6.25, 12.5, 25, 50, 100, 200, and 400 µg·L^−1^) and then passed through a 0.2 μM filter. Doxorubicin was used as a positive standard in all experiments, and at the end of the exposure time, cell growth was analyzed using the MTT assay as described previously [24]. In brief, 15 µL of the MTT stock solution (Promega) (5 mg·L^−1^ in sterile phosphate-buffered saline, pH 7.4) was added to each well after 72 h of incubation of the test extract with the cells. The cells were then incubated for 3 h in the presence of MTT, and then 100 µL of the solubilizing stop solution was added to each well to solubilize the dark violet formazan crystals. Absorbance was measured at 570 nm using a microplate reader (BioteK, USA). Cell viability (Cv%) was determined as in the following equation: Cv (%) = (At/Ac)100, where At and Ac are the absorbances of the test sample and control (blank solvent), respectively. The concentration of the extract that inhibits 50% of the cellular growth (IC_50_) was estimated by least squares regression analysis of a dose-response curve plotting between the percent of cellular inhibition and extract concentrations.

## 3. Results and Discussion

### 3.1. C. gileadensis Callus Culture 

**Figure 1:**
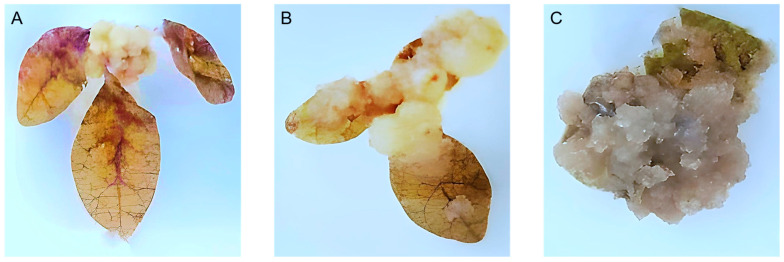
Callus induction of *C. gileadensis* from leaf explant on MS media supplemented with 24.50 μM IBA and 2.22 μM BAP; (**A**) after four weeks in culture; (**B**) after six weeks in culture; (**C**) after eight weeks in culture.

A full callus induction of *C. gileadensis* (100%) was observed on MS media supplemented with 24.50 μM IBA and 2.22 μM BAP (thereafter named “callus induction media”), and the produced callus was friable and lightly creamy in color (Figure 1). On this medium, explants showed inflation and swelling after four weeks of culture in the upper region of the petiole at the junction where it is attached to the leaf blade (Figure 1A). After six weeks of culture, the produced callus proliferated, showing white coloration, a friable texture, and a continuous growth pattern (Figure 1B). After eight weeks of culture, proliferated callus covered the whole explants, and the resulting callus was then subcultured on the same medium until further use (Figure 1C). On the other hand, clear variations in callus induction percentages (less than 20%) were observed between tested treatments, and they rarely formed any viable calli that turned brown and subsequently died. 

**Figure 2:**
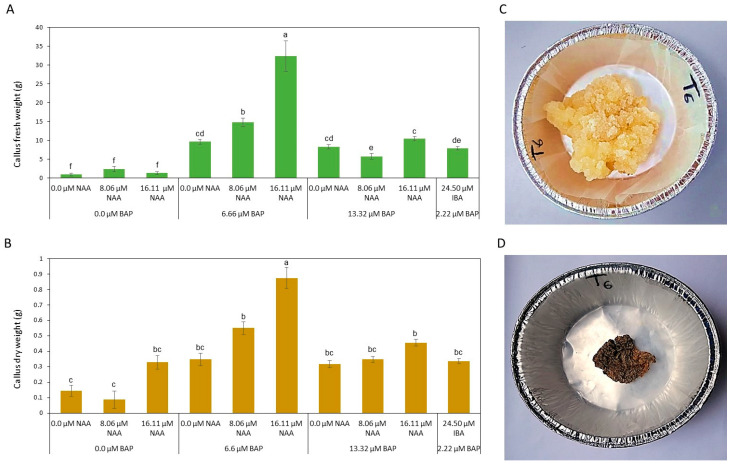
*Commiphora gileadensis* callus growth and proliferation on MS media supplemented with different combinations of plant growth regulators; (**A**) Callus fresh weights (g) after eight weeks of culture on MS media supplemented with different combinations of plant growth regulators; (**B**) Callus dry weights (g) after eight weeks of culture on MS media supplemented with different combinations of plant growth regulators; (**C**) Fresh callus after eight weeks of culture on MS media supplemented 16.11 μM NAA and 6.66 μM BAP; (**D**) Dry callus after eight weeks of culture on MS media supplemented 16.11 μM NAA and 6.66 μM BAP. The mean values (± standard errors) with different letters are significantly different according to Tukey’s HSD test at a probability level of 0.05.

The effect of different NAA and BAP combinations on the growth of *C. gileadensis* callus was investigated. The ANOVA for average callus fresh and dry weight showed a high level of significance (*p* < 0.05) for the different treatments. MS media supplemented with 16.11 μM NAA in combination with 6.66 μM BAP (thereafter named “callus maintenance media”) was the best treatment in promoting *C. gileadensis* callus growth and proliferation after eight weeks in culture, as reflected in producing significantly the highest mean values for callus fresh weight (32.41 g) and dry weight (0.89 g) when compared to all other treatments (Figure 2). On the other hand, the lowest callus fresh weight was obtained by MS media supplemented with 0.0 μM BAP (0.96 g) irrespective of NAA concentrations, while the lowest dry weight was obtained on MS media supplemented with 0.0 μM BAP (0.09 g), in combination with 8.06 μM NAA.

In this study, the higher auxin and lower cytokinin levels resulted in increased callus induction in leaf explants, indicating that the ratio of cytokinin and auxin is important for callus induction in *C. gileadensis*. These results are in general agreement with Nikam et al. [25], who used different types and concentrations of auxins and cytokinins for callus induction in *Boswellia serrata* Roxb, which belongs to the same family as *C. gileadensis*. Similarly, Mishra et al. [26] were successful in inducing callus from juvenile explants of *C. wightii* using MS media supplemented with kinetin and 2,4-D either alone or in different combinations, and the maximum callus fresh weight (5.78 g), callus dry weight (0.572 g), and guggulsterone content percentage (0.062%) were obtained on MS media with 22.65 μM 2,4-D and 2.33 μM kinetin. Al Abdallat et al. [27] studied the effect of different combinations of auxins (IBA or NAA) and cytokinins (BAP or kinetin) on *Crataegus azarolus* callus induction, and they found that MS media supplemented with 24.50 μM IBA and 2.22 μM BAP produced the highest callus induction, which is in consistent with the results of the current study.

In this study, 16.11 μM NAA in combination with 6.66 μM BAP resulted in a substantial increase in *C. gileadensis* callus fresh and dry weights, indicating that the adjustment of the proper ratio of cytokinin and auxin was important for callus growth and cell proliferation. These results agree with Xiong et al. [28], where the high concentration of NAA and BAP in combination enhanced callus growth in *Cordia subcordata*. In addition, high NAA concentrations were used successfully in promoting callus growth in *Piper longum* [29]. In another study, Daffalla et al. [30] found that a combination of 10.74 μM NAA with 6.66 μM BAP increased the callus growth of *Grewia tenax,* and this is in general agreement with the results of this study.

### 3.2. C. gileadensis Cell Suspension Culture 

**Figure 3:**
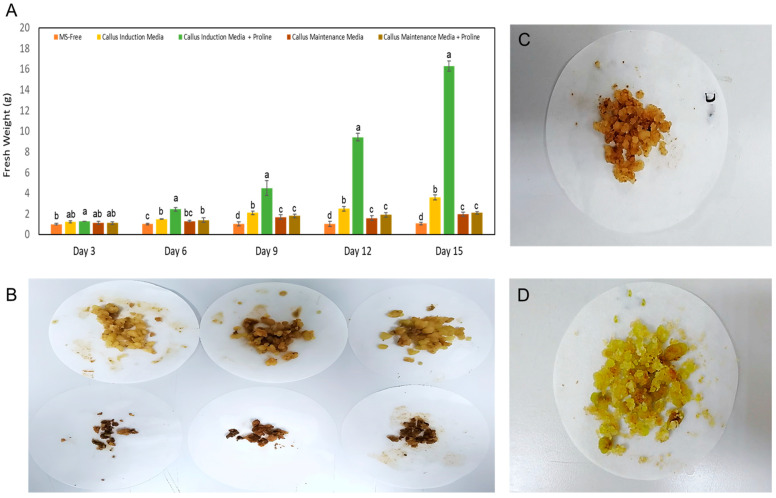
Cell suspension culture of *C. gileadensis*; (**A**) Cells fresh weights (g) measured every three days of culture on different media types; (**B**) Cells suspension cultures after two weeks of culture on MS media supplemented with 24.50 μM IBA and 2.22 μM BAP + 3.0 mg·L^−1^ proline (upper panel) and MS media supplemented with 24.5 μM IBA and 2.22 μM BAP without proline (lower panel); (**C**) Cells suspension cultures after three weeks of culture on MS media supplemented with 24.50 μM IBA and 2.22 μM BAP + 3.0 mg·L^−1^ proline; (**D**) Cells suspension cultures after five weeks of culture on MS media supplemented with 24.50 μM IBA and 2.22 μM BAP + 3.0 mg·L^−1^ proline. The mean values (± standard errors) with different letters are significantly different according to Tukey’s HSD test at a probability level of 0.05.

In this study, *C. gileadensis* cell suspension culture was established successfully using the callus induction media (24.50 μM IBA + 2.22 μM BAP) either supplemented with or without 3.0 mg·L^−1^ proline (Figure 3). However, the callus induction media supplemented with 3.0 mg·L^−1^ proline was more effective in promoting cell growth, which resulted in a substantial increase in fresh weight that started after two weeks of culture and continued to grow up to six weeks (Figure 3). Callus maintenance media with or without 3.0 mg·L^−1^ proline failed to promote the growth of cells and the callus inoculum turned dark brown and subsequently cell death was observed after two weeks in culture. 

In this study, the production of *C. gileadensis* cell suspension culture from callus explants was achieved successfully using callus induction media, which was found suitable for cell culture in liquid forms. Furthermore, the addition of 3.0 mg·L^−1^ proline to callus induction medium boosted the production of viable cells compared with other treatments. Previously, *C. wightii* cell suspension culture was established successfully using MS media containing 2.27 μM 2,4-D in combination with 1.16 μM kinetin [31], indicating the need for a high auxin to cytokinin ratio to promote cell growth, and this is in general agreement with the results of this study. Similar to our results, the cell suspension culture of *Azadirachta indic* was obtained using half MS medium supplemented with 4.9 μM IBA and 2.22 μM BAP [32]. The *C. wightii* cell suspension culture was used successfully to boost the production of guggulsterone, a valuable secondary metabolite obtained from the gum resin of the tree [33] indicating the possibility of using *C. gileadensis* cell suspension culture for secondary metabolite production. 

Proline is one of the amino acids and considered a major constituent of cellular proteins and it has various roles in plants under normal and stress conditions and it has a notable effect on plant growth and development [34]. The addition of proline in cell suspension culture was found to have a promoting effect on cell growth and the maintenance of friable and embryogenic callus in different plant species [34]. In this study, proline had a positive and promoting effect on the growth and proliferation of the cells as compared to other treatments. The exogenous application of proline was found to increase rice cell growth and extended viability, which is in general agreement with the results of this study [35].

### 3.3. Antimicrobial Effects of C. gileadensis Methanolic Extract

The antimicrobial results of different *C. gileadensis* methanolic extracts against selected pathogenic bacteria and *Candida albicans* are shown in Table 1. Out of eight microbes, no clear antimicrobial effects of *C. gileadensis* methanolic extracts were observed on *E. coli*, *E. carotovora,* and *K. pneumonia* (Table 1). On the other hand, *S. aureus* was the most affected microbe, with all methanolic extracts showing clear antimicrobial effects on its growth except for the callus extract. The highest zone of inhibition for *S. aureus* was 2.5 cm, which was obtained with the leaf extract (Table 1). The leaf extract was also effective in inhibiting the growth of *S. epidermidis*, *B. subtilis*, *Salmonella,* and *C. albicans*. The callus methanolic extract showed no growth inhibitory effect on all tested microbes, while the cell suspension culture was effective against *S. epidermidis* and *S. aureus* (Table 1). 

### 3.4. Cytotoxic Effects of C. gileadensis Methanolic Extract

The cytotoxicity of different methanolic extracts of *C. gileadensis* was evaluated against the proliferation of different cancer lines, PanC1, A549, MCF7, and PC3, which represent different cancer types: pancreatic, lung, breast, and prostate cancer, respectively. The cytotoxicity of methanolic extracts was compared to normal human fibroblast cell lines. Among all cell lines investigated, only A549 was sensitive to all extracts, indicating the selective activity of these extracts against a specific cell line (Table 2). In addition, leaf extract showed the strongest cytotoxicity against A549 and was also effective against all tested cell lines, but it also showed clear toxicity against Fibroblast cells (Table 2). Besides the A549 cell line, callus methanolic extract showed weak cytotoxicity against the PanC1 cell line. The seed methanolic extract was more effective against the PanC1 cell line compared to the callus methanolic extract, with no effect against them using the cell suspension methanolic extract (Table 2). The cytotoxic activity of the *C. gileadensis* extracts against cancer cell lines observed in this study is in general agreement with several previous reports that demonstrated multiple pharmacological activities that support its potential cytotoxic activity [8,10,11]. The specific cytotoxicity against the A549 cell line observed in this study was reported previously in several *Commiphora* related species, such as *Resina Commiphora* [36] and *C. africana* [37].

### 3.5. Secondary Metabolites Profiling of C. gileadensis 

The LC-MS/MS analysis of the methanolic extracts was subjected to a computational analysis approach using GNPS. The mass spectral molecular networking analysis has revealed the presence of various natural products in different extracts of *C. gileadensis*, and some of them were identified exclusively in callus or cell suspension culture extracts (Table 3). Representative Total Ion Chromatogram (TIC) of the different *C. gileadensis* extracts are available in the supplementary data (Figures S1–S4). 

**Figure 4:**
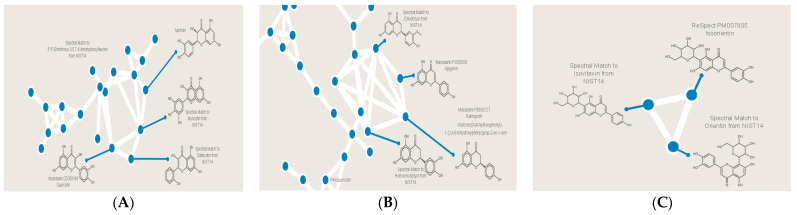
Molecular families of *C. gileadensis* methanolic extracts identified using the GNPS approach: (**A**) flavonols; (**B**) flavonones; (**C**) flavonoids glycosides.

As expected, one of the most abundant molecular families is the one that contains several natural products, which belong to the subclass flavanonols in the flavonoids group (Figure 4A). The nodes in this molecular family were annotated with GNPS community-contributed library hits to identify several natural products in this family, like taxifolin, myricetin, datiscetin, and quercetin. These are all related compounds with similar structural cores, which explains their clustering into one molecular family. The largest molecular family in this network was characterized as containing mostly falvonones like eriodictyol, homoeriodictyol, apigenin, and naringenin (Figure 4B). These compounds are structurally similar, and this was reflected in the family, where they were strongly connected. However, in the outer edges of the same molecular family, more compounds were identified, like pinoquercetin, laudanosine, trans-resveratol, and catechin gallate. While these molecules are not classified as falvonones, they share many similarities in their core structural features with this group. Another family was identified that contains flavonoid glycosides, in which the nodes were annotated as isovitexin, isoorientin, and orientin (Figure 4C). 

**Figure 5:**
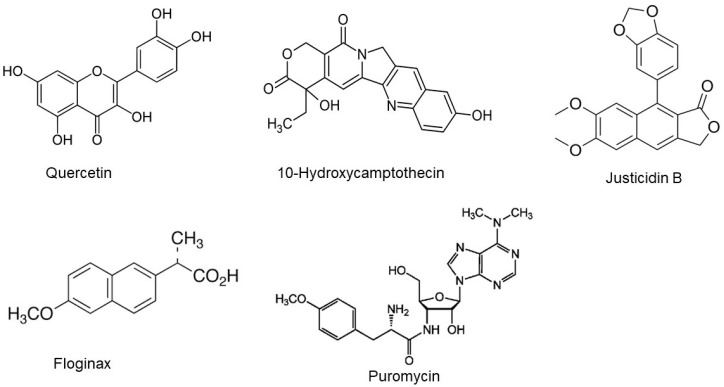
Selected molecules identified in *C. gileadensis* methanolic extracts using the GNPS approach.

Other glycoside derivatives of the flavonoid quercetin were also observed, but they were not grouped into one family, likely due to structural differences in their sugar moiety (Figure 5). Two uncommon families were also observed in the extracts: the first one includes 10-hydroxycamptothecin, justicidin B, and floginax (Figure 5), while the second family contains molecules that are likely to belong to bacterial secondary metabolites (Figure 5). This interesting family includes puromycin, an aminonucleoside antibiotic that originated from the *Streptomyces alboniger* bacterium. Putative assignments were inspected and confirmed using manual inspection of raw mass spectrometry data, fragmentation patterns, and retention time analysis, which further validated the discovery of this molecular family.

The LC-MS analysis of *C. gileadensis* extracts revealed the presence of several important phytochemical compounds with cytotoxic activities like eriodictyol, homoeriodictyol, apigenin, naringenin, pinoquercetin, laudanosine, esveratrol, catechin isovitexin, isoorientin, orientin, and quercetin, which are known to have anti-proliferative activities [9,10,24,38,39]. Thus, the antimicrobial and cytotoxic effects demonstrated for *C. gileadensis* extracts can be attributed, at least in part, to the presence of these compounds. However, the discrepancies in the cytotoxic and antimicrobial potencies of these extracts can be correlated to the higher concentrations of the secondary metabolites that were detected in the leaf extract. Moreover, the detection of some secondary metabolites in leaf extracts only, like pinoquercetin, catechin gallate, quercetin and 10-Hydroxycamptothecin, could also justify the obvious cytotoxic effects of leaf extracts against the tested cell lines. However, further quantitative targeted analyses are required to quantify the precise concentration of each secondary metabolite. Finally, *C. gileadensis* callus and cell suspension cultures established in this study proved to be useful for the production of secondary metabolites, and such systems can be used to boost their production as observed in other plant species [40,41].

## 4. Conclusions

In the current study, *C. gileadensis* callus and cell suspension cultures were established successfully using mature leaves as explants. Callus was induced successfully on MS media supplemented with 24.50 μM of IBA and 2.22 μM BAP, and the same media supplemented with 3.0 mg·L^−1^ proline were found to be the best media to support optimal growth of cell suspension cultures. The 100% success rate of callus induction, optimal growth of callus and cell suspension cultures observed in this study can be utilized for the mass production of secondary metabolites with pronounced antimicrobial and cytotoxic effects. Furthermore, it is considered a promising approach to alleviating the pressure on endangered plants, such as *C. gileadensis*, in their natural habitats. The LCMS-GNPS analyses proved to be a powerful tool to dissect the chemical constituents of *C. gileadensis* extracts. Several metabolites were identified in *C. gileadensis* methanolic extracts, including flavonols, flavanones, and flavonoid glycosides. Interestingly, *C. gileadensis* leaf extract was effective in inhibiting the growth of a wide range of bacterial species, while cell suspension culture was found to be effective against *S. epidermidis* and *S. aureus*. Similarly, *C. gileadensis* leaf extract showed a broad cytotoxicity effect against several tested cancer cell lines, while callus and cell suspension cultures showed a specific cytotoxic effect against A549 cell lines. Further studies are required to isolate and identify the most active constituents and to explore the growth environments required to enrich the in vitro production of these active constituents. 

## Figures and Tables

**Table 1:** Antimicrobial effects of different methanolic extracts of *C. gileadensis* using the disc diffusion (IZT) and the minimum inhibitory concentration (MIC) assays.

	Callus		Cell Suspension		Leaves		Seeds		Tetracycline	
Microbial Species	IZT ^y^	MIC ^Z^	IZT	MIC	IZT	MIC	IZT	MIC	IZT	MIC
*B. subtilis*	-	-	-	-	-	6.25	-	-	18.0 ± 0.20	1.56
*E. coli*	-	-	-	-	-	-	-	-	10.06 ± 0.17	0.39
*S. aureus*	-	-	-	6.25 *	2.50 ± 0.07 **	6.25	2.30 ± 0.17	1.56	17.00 ± 0.10	0.78
*S. epidermidis*	-	-	-	6.25	1.50 ± 0.09	3.12	-	3.12	14.00 ± 0.84	1.56
*Salmonella* spp.	-	-	-	-	1.70 ± 0.14	12.50	-	12.50	22.20 ± 0.66	0.78
*E. carotovora*	-	-	-	-	-	-	-	-	18.00 ± 0.20	1.56
*K. pneumoniae*	-	-	-	-	-	-	-	-	17.00 ± 0.10	1.56
*C. albicans*	-	-	-	-	-	6.25	-	-	18.00 ± 0.00	6.25

* Concentration in mg·L^−1^; ** Mean value ± S.E; ^y^ IZT: the diameter of the inhibition zone of the test sample at a given concentration; ^Z^ MIC Minimum inhibitory concentration.

**Table 2:** Cytotoxic effects of different methanolic extracts of *C. gileadensis* against selected cancer cell lines.

Cell Line	Callus	Cell Suspension	Leaves	Seeds	Doxorubicin
PanC1	488.20 * ± 25.40 **	NA	13.60 ± 1.20	106.6 ± 6.7	1.30 ± 0.09
A549	117.50 ± 3.50	137.40 ± 9.90	1.00 ± 0.50	148.7 ± 12.4	3.50 ± 0.03
MCF7	NA ***	NA	34.20 ± 2.80	350.00 ± 32.50	5.00 ± 0.79
PC3	NA	NA	76.40 ± 4.70	NA	1.10 ± 0.05
Fibroblast	Not toxic	Not toxic	82.50 ± 7.90	Not toxic	0.022 ± 0.00

* IC_50_ is defined as the concentration (μg/mL) at which 50% of cell viability was inhibited; ** Mean value ± S.D, *** NA: Not Applicable.

**Table 3:** Examples of detected secondary metabolites in different methanolic extracts of *C. gileadensis* by using LC-MS/MS analysis.

Compound Name	Retention Time	Precursor m/z [M + H]+	Molecular Formula	Plant Extract
Eriodictyol	14.82	287.15	C_15_H_12_O_6_	Callus and cell suspension
Homoeriodictyol	13.91	303.10	C_16_H_14_O_6_	Cell suspension
Apigenin	13.65	271.20	C_15_H_10_O_5_	Cell suspension
Naringenin	13.55	273.12	C_15_H_12_O_5_	Cell suspension
Pinoquercetin	5.80	317.02	C_16_H_12_O_7_	Leaves
Laudanosine	7.81	358.13	C_21_H_27_NO_4_	Cell suspension, seeds and leaves
Catechin Gallate	12.51	443.12	C_22_H_18_O_10_	Leaves
Isovitexin	9.52	433.10	C_21_H_20_O_10_	Cell suspension
Isoorientin	11.42	449.08	C_21_H_20_O_11_	Cell suspension
Orientin	9.35	449.06	C_21_H_20_O_11_	Cell suspension
Quercetin	4.96	303.05	C_15_H_10_O_7_	Leaves
10-Hydroxycamptothecin	3.74	365.15	C_20_H_16_N_2_O_5_	Leaves
Justicidin B	16.91	365.15	C_21_H_16_O_6_	Callus, cell Suspension and leaves
Floginax	16.15	230.96	C_14_H_14_O_3_	Callus and leaves
Puromycin	8.32	472.17	C_22_H_29_N_7_O_5_	Callus, cell Suspension, seeds and leaves

## Data Availability

Data is not publicly available due to privacy or ethic restriction. The datasets supporting the results of this article will be freely available upon reasonable request from Yasser Bustanji and Ayed M. Al-Abdallat.

## Supplementary Materials

The following supporting information can be downloaded at: https://www.mdpi.com/article/10.3390/metabo13040537/s1, Figure S1. TIC chromatogram for the extract of the callus. Figure S2. TIC chromatogram for the extract of the cell suspension. Figure S3. TIC chromatogram for the extract of the leaves. Figure S4. TIC chromatogram for the extract of the seed
