# Supplementary material for: Secondary Metabolites Profiling, Antimicrobial and Cytotoxic Properties of Commiphora gileadensis L. Leaves, Seeds, Callus, and Cell Suspension Extracts
Source: Metabolites. 2023 Apr 10;13(4):537. doi: 10.3390/metabo13040537 (PMC10146941; doi:10.3390/metabo13040537)
Supplement: Supplementary file 1 [file metabolites-13-00537-s001.zip › metabolites-2105495-supplementary.pdf]

Supplementary Data

The following is the Total Ion Chromatogram (TIC) of the different extracts:

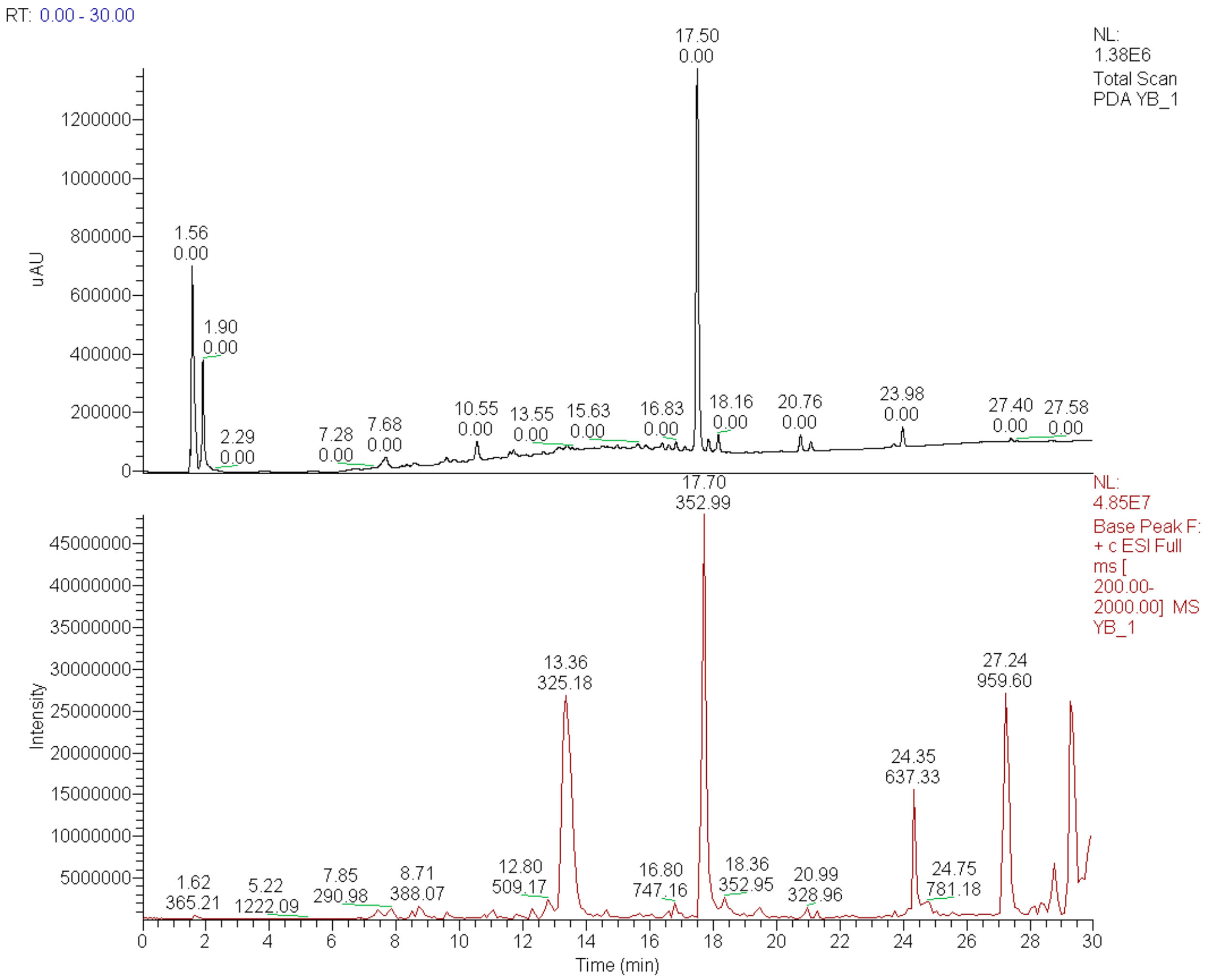

Figure S1. TIC chromatogram for the extract of the callus

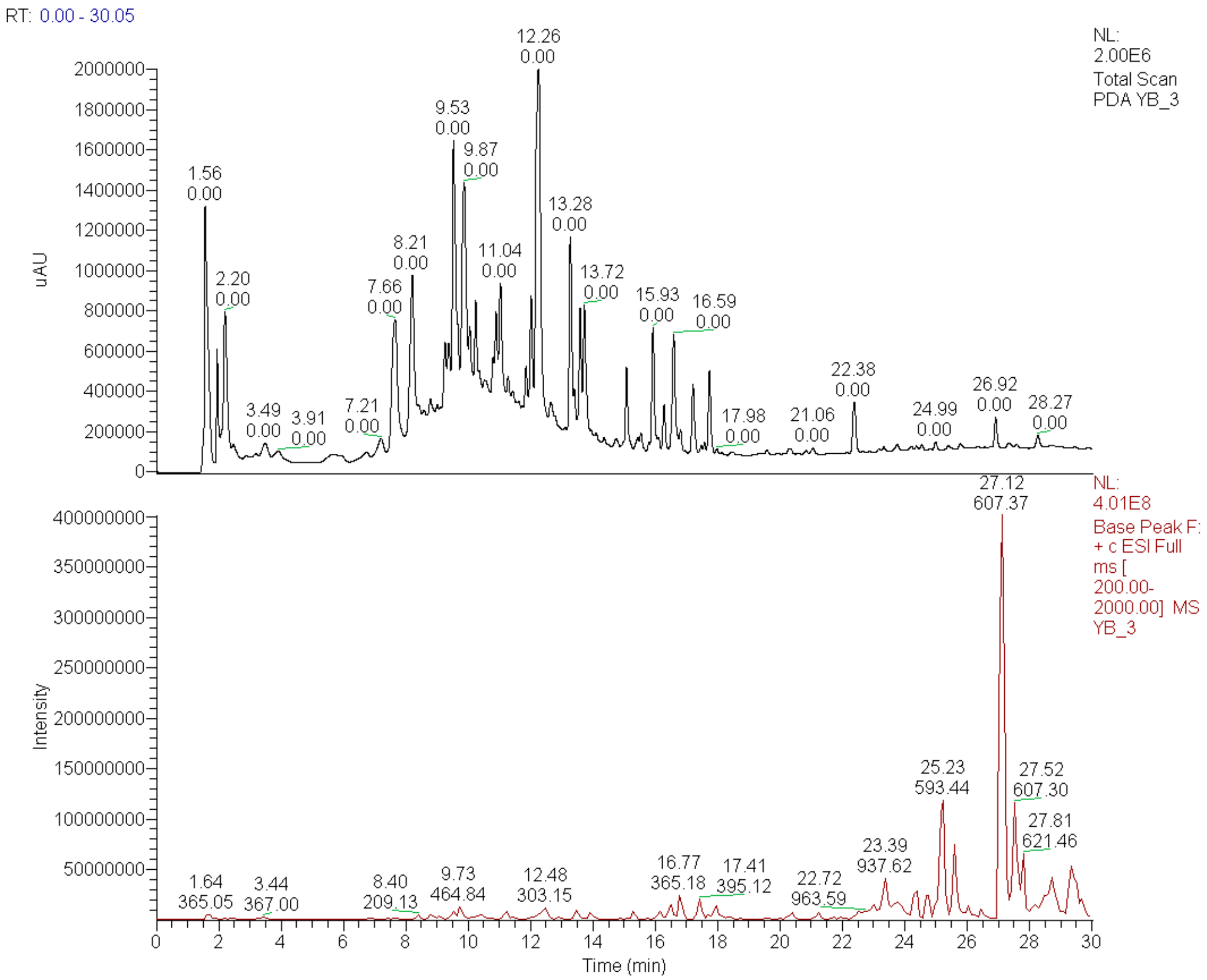

Figure S2. TIC chromatogram for the extract of the cell suspension

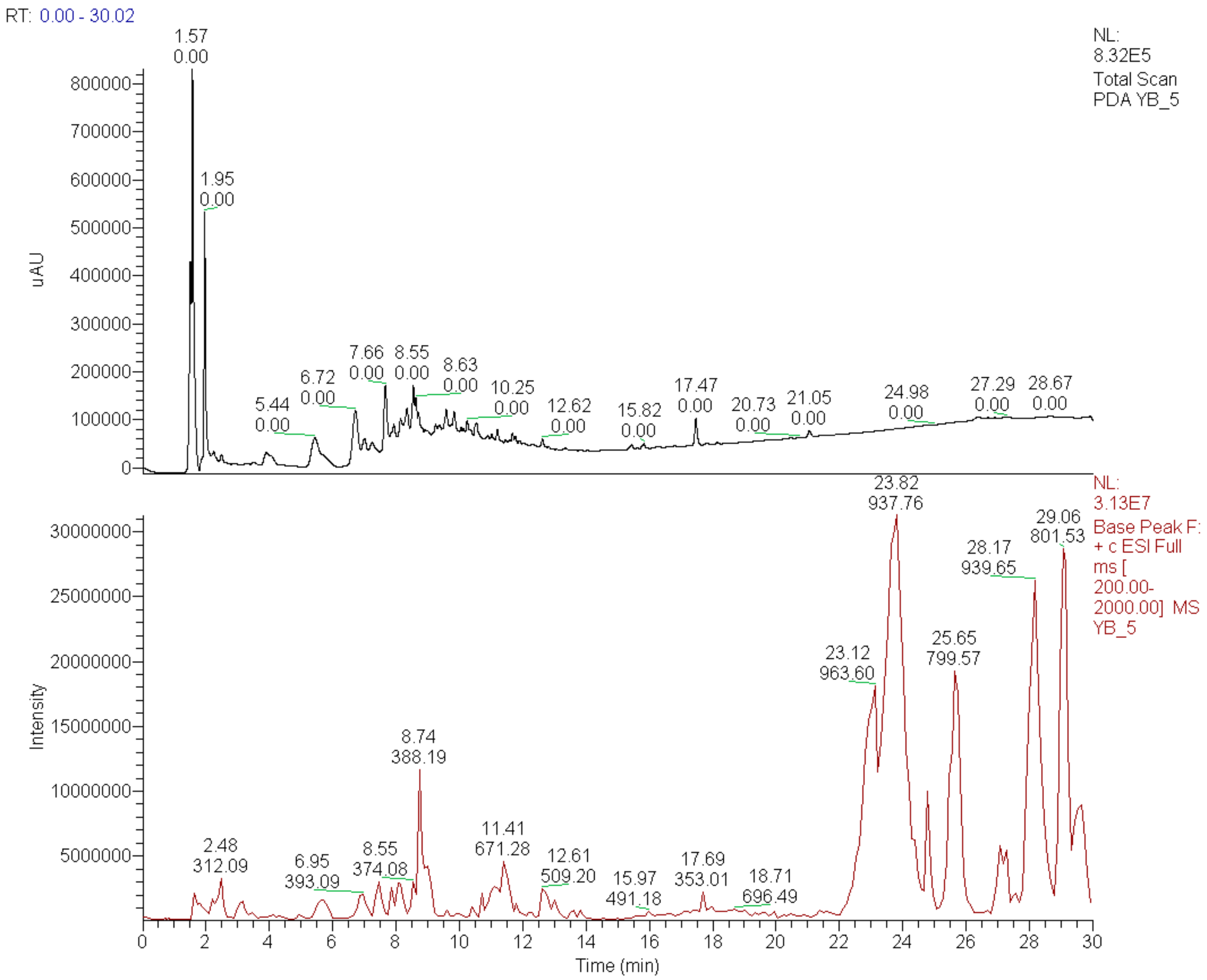

Figure S3. TIC chromatogram for the extract of the leaves

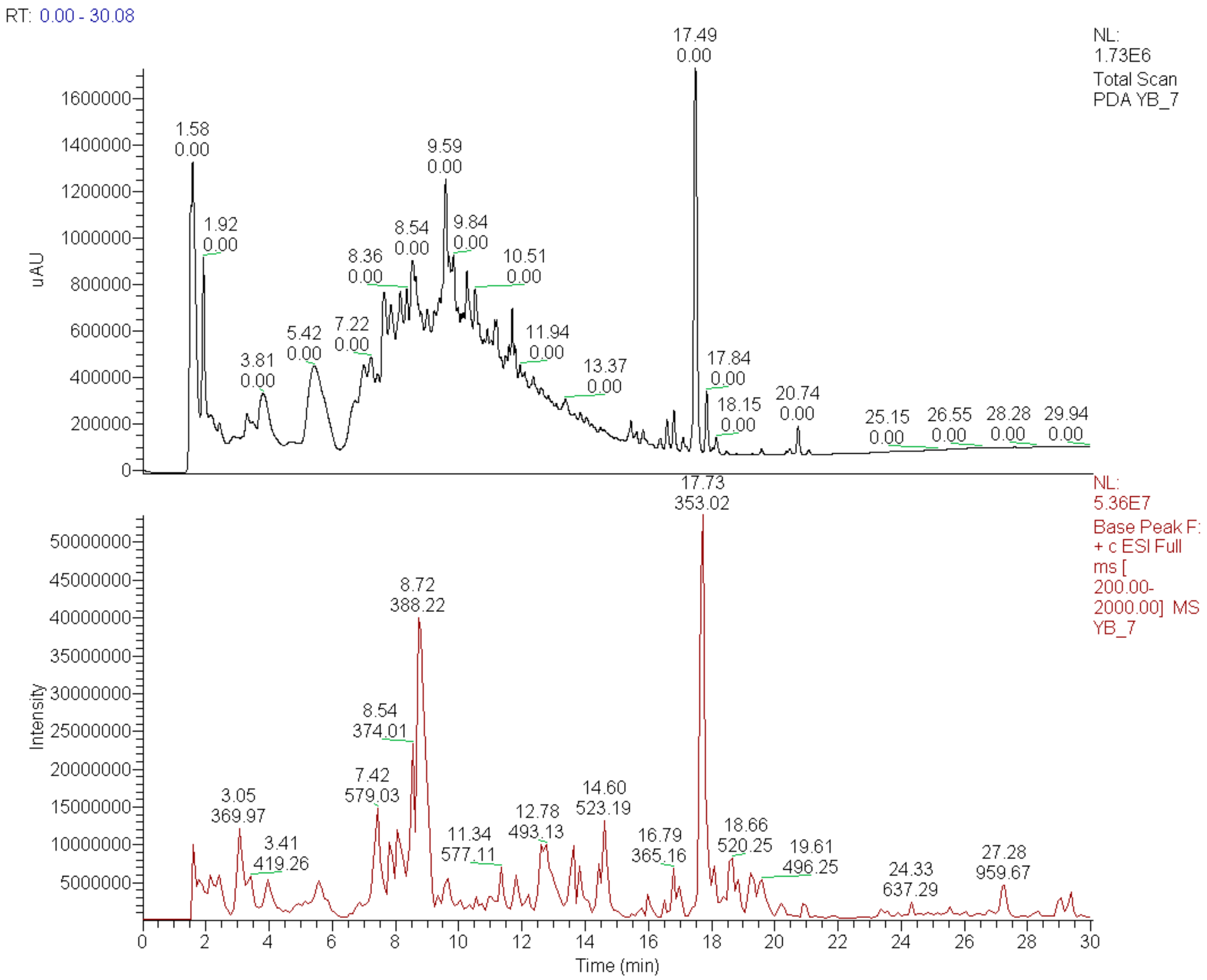

Figure S4. TIC chromatogram for the extract of the seed
